# Editorial: Unconventional roles of endothelial cells

**DOI:** 10.3389/fcell.2024.1439419

**Published:** 2024-07-08

**Authors:** Lorenzo Iovino, Guido Krenning, Brandon Hadland

**Affiliations:** ^1^ Fred Hutchinson Cancer Center, Seattle, United States; ^2^ University Medical Center Groningen, Groningen, Netherlands

**Keywords:** endothelial cells (EC), endothelium, regenerative medicine, tumor microenvironment (TME), vasculature

## Abstract

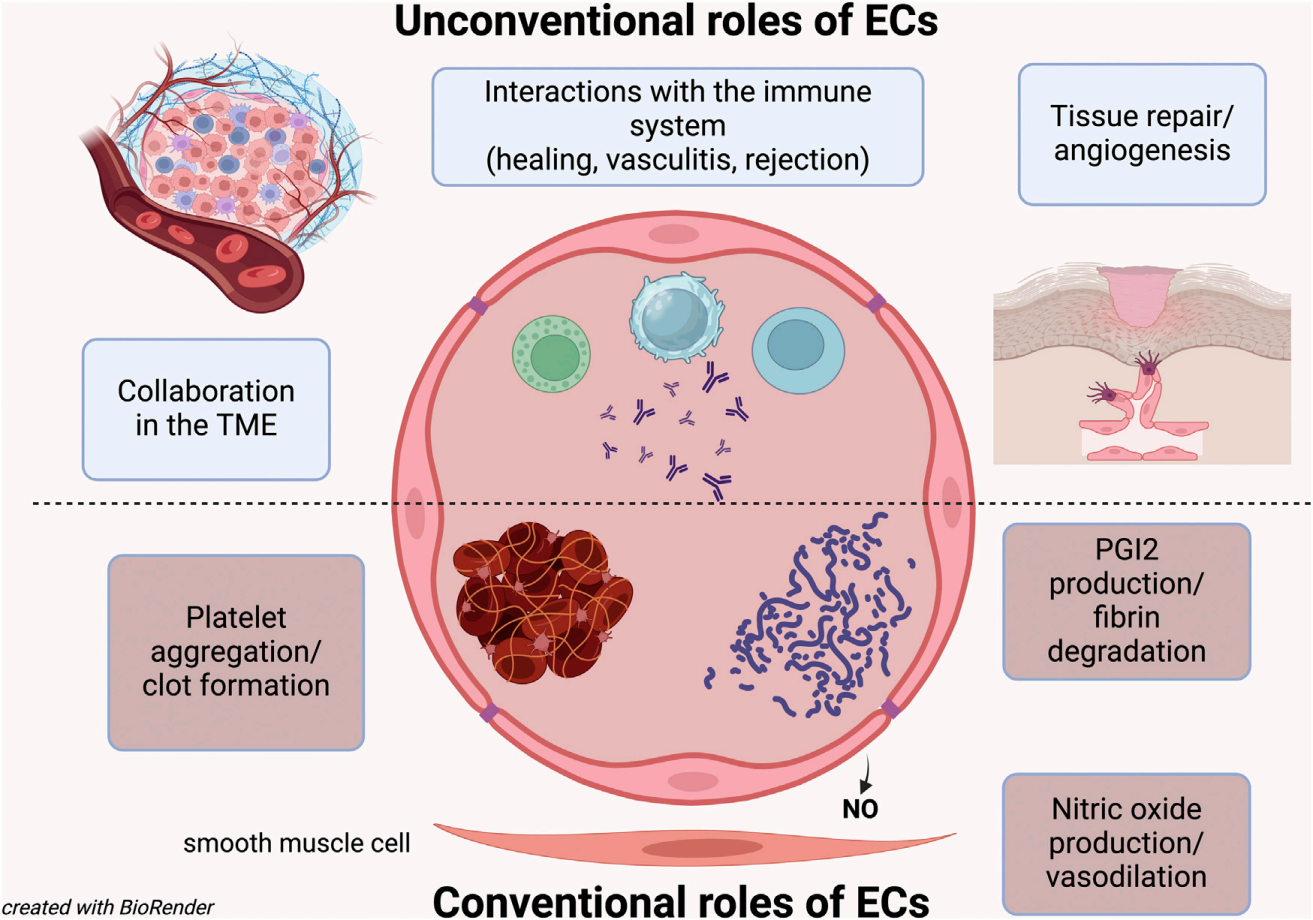

Contrary to common knowledge and what is often taught in medical school, the largest organ in our body is not the skin, which can reach a surface area of 2 square meters. Nor is it the intestine, the longest organ, which covers approximately 32 square meters (Helander and Fandriks, 2014; Allaire et al., 2018). The largest organ is actually the endothelium: composed of a single layer of cells of varying shapes and sizes, the endothelium consists of approximately 10–60 trillion endothelial cells (Aird, 2005; Rajala, 2021), covering a surface area ranging from 300 to 1000 square meters. To put this into perspective, that’s the size of a large house or one to two basketball courts.

Endothelial cells (ECs) form the thin layer lining all blood vessels that constitues the endothelium; originating from the mesoderm (Aquino et al., 2021; Hou et al., 2022), they serve as the first barrier between the bloodstream and tissues, and their primary function is to regulate the exchange between these two compartments.

For years, the only role attributed to the ECs was to trigger platelet aggregation and activate coagulation factors (Wu et al., 1988; Ruggeri, 2003). However, it was later discovered that they also play a crucial role in producing anti-aggregant factors and preventing unnecessary clots (Preissner, 1988). While this coagulation balance role was long considered their only function, it is now understood that being the endothelium such a vast organ, with ECs being anatomically and functionally different depending on the type of vessel (arteries, capillaries, veins) and organ (Hou et al., 2022; Wakabayashi and Natio), must have additional roles.

We now know that ECs help regulate blood flow to individual organs and different regions (Clifford, 2011; Ashby and Mack, 2021), and they also play a significant role in blood pressure regulation (Clifford, 2011). Moreover, we know that many vascular diseases are caused by endothelial dysfunction or by ECs being targeted by autoimmune phenomena or infections (Pall and Savage, 1994; Gobel et al., 1996; Nakaoka et al., 2018; Xu et al., 2022; Laan et al., 2024).

Thus, beyond their classical functions, ECs are now recognized for their role in tissue regeneration (Moonen et al., 2010; Hadland et al., 2022), cancer biology (with both pro- and anti-tumor effects), and immune regulation (Fang et al., 2023).

This Research Topic aims to explore some of these unconventional aspects of EC biology.

Wakabayashi and Natio cover the contribution of organ-specific ECs studied with single cell RNA sequencing: the study highlights the heterogeneity of ECs and identifies specific molecular signatures and functional properties unique to ECs from various tissues. By analyzing the transcriptional profiles at the single-cell level, the researchers were able to map out the diverse landscape of EC subtypes and their respective roles in organ-specific physiology and pathology. This comprehensive analysis enhances our understanding of the specialized functions of ECs in different organ systems and provides a valuable resource for future research in vascular biology and related therapeutic interventions.

The article by Ribatti and D’Amati delves into the mechanisms and pathways of angiogenesis specifically within bone tissue. It explores the critical mediators involved in the formation of new blood vessels in the bone, including growth factors like VEGF (vascular endothelial growth factor), FGF (fibroblast growth factor), and angiopoietins. The authors discuss the complex interplay between these mediators and various signaling pathways that regulate bone angiogenesis. The review highlights how these processes are essential for bone development, repair, and remodeling, and also considers the implications for pathological conditions such as bone tumors and osteoporosis. The detailed examination of angiogenic mediators and pathways provides insights into potential therapeutic targets for bone-related diseases.

The article by Allbritton-King and García-Cardeña addresses the significant and evolving role of endothelial cells (ECs) in cardiac disease. It highlights how EC dysfunction contributes to various cardiovascular conditions, such as atherosclerosis, hypertension, and heart failure. The authors discuss the molecular and cellular mechanisms underlying EC dysfunction, including the impact of inflammation, oxidative stress, and metabolic disturbances. They also explore recent advancements in understanding the interactions between ECs and other cell types within the heart, and how these interactions influence cardiac health and disease progression. The article emphasizes the potential for targeting ECs in therapeutic strategies to treat or prevent cardiac disease, noting the promise of novel interventions aimed at restoring EC function and improving cardiovascular outcomes. This comprehensive review underscores the critical role of ECs in cardiac pathophysiology and the importance of ongoing research in this area.

Finally, the article by Xu et al. explores the pivotal role of endothelial cells (ECs) within the tumor microenvironment. The authors examine how ECs contribute to tumor growth, metastasis, and the formation of new blood vessels (angiogenesis) which supply nutrients and oxygen to the tumor. The study highlights the interactions between ECs and tumor cells, including the signaling pathways and molecular mechanisms that facilitate these interactions. also discuss how ECs help create an immunosuppressive environment, protecting the tumor from the body’s immune response. The article reviews therapeutic strategies targeting ECs to inhibit tumor angiogenesis and improve cancer treatment outcomes, emphasizing the potential of anti-angiogenic therapies and their impact on the tumor microenvironment. This comprehensive analysis underscores the complex role of ECs in cancer biology and their significance as a target for innovative cancer therapies.

These significant papers address only a portion of the numerous unresolved questions regarding the unconventional roles of ECs. For instance, we have yet to fully understand how EC turnover is regulated homeostatically; this question becomes even more critical in clinical contexts that require tissue regeneration and revascularization. Additionally, the origin of ECs is still debated: under certain circumstances, do mesenchymal cells, fibroblasts, hematopoietic stem cells, or tissue-resident stem cells serve as sources of ECs (Moonen et al., 2010; Aquino et al., 2021; Trimm, 2023)?

In the context of organ and bone marrow transplants, where the endothelium is the first interface between donor and recipient immune cells, is it possible to prevent organ rejection and/or graft-versus-host disease by selectively targeting endothelial sites of immune-EC interactions? Notably, in bone marrow transplants, some of the most lethal complications are endothelial-related, such as vein occlusive disease of the liver/sinusoid occlusion syndrome (VOD/SOS), diffuse alveolar hemorrhage (DAH), and transplant-associated thrombotic microangiopathy (TA-TMA) (Luft et al., 2021).

Further basic science studies are essential to provide crucial answers and identify novel targets to prevent and treat these conditions. This research will simultaneously offer deeper insights into harnessing the power of ECs in regenerative medicine, anti-tumor therapies, and immunology.
